# Supplementary material for: MicroRNA-934 is a novel primate-specific small non-coding RNA with neurogenic function during early development
Source: eLife. 2020 May 27;9:e50561. doi: 10.7554/eLife.50561 (PMC7295570; doi:10.7554/eLife.50561)
Supplement: Supplementary file 1. [file elife-50561-supp1.docx]

**Supplemental Table 1.** The 144 miRNAs across all differentiation stages demonstrating tissue specificity index tau > 0.7.

| **miRNAs** | **Tau** | **Fibroblasts**  **Median** | **iPSCs**  **Median** | **NPCs**  **Median** | **Neurons**  **Median** |
| --- | --- | --- | --- | --- | --- |
| **hsa-let-7a-2-3p** | 0,729512974 | 4,392534415 | 0,000144262 | 0,000144262 | 1,87308662 |
| **hsa-miR-100-3p** | 0,72260882 | 1,713094706 | 0,000144262 | 0,000144262 | 0,918513358 |
| **hsa-miR-1185-2-3p** | 0,796151277 | 5,778517556 | 1,020829652 | 0,000144262 | 1,646520008 |
| **hsa-miR-1185-5p** | 0,893035815 | 2,74339306 | 0,000144262 | 0,000144262 | 0,000144262 |
| **hsa-miR-122-3p** | 0,797027672 | 0,000144262 | 0,733891745 | 1,882531548 | 0,000144262 |
| **hsa-miR-1224-5p** | 0,726183601 | 0,000144262 | 0,733891745 | 0,792817883 | 2,241468612 |
| **hsa-miR-1245a** | 0,786595076 | 1,097183874 | 0,000144262 | 2,672273716 | 0,619583581 |
| **hsa-miR-1263** | 0,739164771 | 0,000144262 | 5,003350644 | 0,922810582 | 0,000144262 |
| **hsa-miR-1269b** | 0,807804654 | 0,000144262 | 1,455711033 | 1,360669755 | 0,41465712 |
| **hsa-miR-1272** | 0,801973595 | 0,000144262 | 2,21620858 | 0,000144262 | 0,000144262 |
| **hsa-miR-1283** | 0,706766686 | 0,000144262 | 4,45568539 | 2,29864637 | 0,41465712 |
| **hsa-miR-1293** | 0,804038374 | 0,000144262 | 0,714405852 | 1,481045729 | 0,000144262 |
| **hsa-miR-1323** | 0,710283553 | 0,000144262 | 6,288617174 | 2,088047528 | 0,805256779 |
| **hsa-miR-141-3p** | 0,707902639 | 0,668191606 | 3,657512058 | 3,023476681 | 0,805256779 |
| **hsa-miR-141-5p** | 0,94096075 | 0,000144262 | 0,660872993 | 0,000144262 | 0,000144262 |
| **hsa-miR-144-3p** | 0,714750367 | 0,000144262 | 0,754246778 | 1,301476446 | 0,805256779 |
| **hsa-miR-153-3p** | 0,791475674 | 0,000144262 | 0,714405852 | 0,83517372 | 2,669801534 |
| **hsa-miR-153-5p** | 0,900365234 | 0,000144262 | 0,000144262 | 0,000144262 | 1,051555365 |
| **hsa-miR-154-3p** | 0,719980519 | 5,229466784 | 2,448646511 | 1,370052327 | 1,084836566 |
| **hsa-miR-183-3p** | 0,719381508 | 0,000144262 | 2,356145028 | 2,862327364 | 0,000144262 |
| **hsa-miR-187-5p** | 0,7791826 | 0,000144262 | 2,368683912 | 0,83517372 | 0,000144262 |
| **hsa-miR-190a-3p** | 0,732146401 | 1,415229491 | 0,714405852 | 1,974353102 | 0,000144262 |
| **hsa-miR-1911-3p** | 0,730666195 | 0,000144262 | 1,697140252 | 0,000144262 | 0,805256779 |
| **hsa-miR-1911-5p** | 0,726318899 | 0,000144262 | 2,86765618 | 0,841932638 | 2,125388436 |
| **hsa-miR-1912** | 0,711925194 | 0,000144262 | 2,537396731 | 0,000144262 | 1,084836566 |
| **hsa-miR-19b-2-5p** | 0,70478756 | 0,000144262 | 0,754246778 | 1,360669755 | 0,000144262 |
| **hsa-miR-200b-5p** | 0,73378169 | 0,000144262 | 1,455711033 | 1,301476446 | 0,000144262 |
| **hsa-miR-208b-3p** | 0,741559272 | 0,000144262 | 0,000144262 | 0,83517372 | 2,583357215 |
| **hsa-miR-218-1-3p** | 0,771911272 | 0,000144262 | 0,000144262 | 2,048231794 | 0,458149299 |
| **hsa-miR-223-3p** | 0,846725148 | 0,000144262 | 1,455711033 | 0,792817883 | 0,000144262 |
| **hsa-miR-224-3p** | 0,73386388 | 3,381806268 | 0,000144262 | 1,301476446 | 0,616140144 |
| **hsa-miR-23a-5p** | 0,739425284 | 3,254035781 | 0,754246778 | 0,858383453 | 0,531202547 |
| **hsa-miR-2682-3p** | 0,760048147 | 2,844865318 | 0,000144262 | 0,83517372 | 1,4138698 |
| **hsa-miR-29b-1-5p** | 0,751297011 | 4,491716126 | 1,547065794 | 0,858383453 | 0,000144262 |
| **hsa-miR-302b-5p** | 0,706690675 | 0,000144262 | 8,084163138 | 1,781912374 | 0,000144262 |
| **hsa-miR-302d-5p** | 0,716246384 | 0,000144262 | 8,067148843 | 2,048231794 | 0,000144262 |
| **hsa-miR-302e** | 0,704641 | 0,000144262 | 6,244982713 | 1,370052327 | 0,000144262 |
| **hsa-miR-302f** | 0,76757423 | 0,000144262 | 2,800594419 | 0,83517372 | 0,000144262 |
| **hsa-miR-3130-5p** | 0,755887857 | 0,874648093 | 0,754246778 | 0,858383453 | 0,619583581 |
| **hsa-miR-3150a-5p** | 0,729414439 | 0,668191606 | 1,410150303 | 1,301476446 | 0,000144262 |
| **hsa-miR-3152-5p** | 0,810334604 | 3,663179613 | 0,42556153 | 0,000144262 | 0,999094788 |
| **hsa-miR-3167** | 0,79080393 | 4,108740659 | 0,000144262 | 0,83517372 | 0,616140144 |
| **hsa-miR-329-5p** | 0,713586621 | 2,3569773 | 5,898505208 | 0,000144262 | 0,000144262 |
| **hsa-miR-337-3p** | 0,821309419 | 6,18696875 | 0,000144262 | 0,000144262 | 0,918513358 |
| **hsa-miR-337-5p** | 0,874099532 | 4,28637744 | 0,000144262 | 0,000144262 | 0,000144262 |
| **hsa-miR-3656** | 0,772391774 | 0,650519567 | 0,660872993 | 0,000144262 | 0,805256779 |
| **hsa-miR-367-5p** | 0,791280687 | 0,000144262 | 4,530102167 | 0,000144262 | 0,000144262 |
| **hsa-miR-370-5p** | 0,850683722 | 3,947676061 | 1,189991938 | 0,000144262 | 0,619583581 |
| **hsa-miR-371a-3p** | 0,756677785 | 0,000144262 | 4,530102167 | 3,010036224 | 0,000144262 |
| **hsa-miR-372-5p** | 0,83305688 | 0,000144262 | 2,767546839 | 0,000144262 | 0,000144262 |
| **hsa-miR-373-3p** | 0,70630481 | 0,000144262 | 6,487648198 | 3,82603895 | 0,000144262 |
| **hsa-miR-376a-3p** | 0,709830809 | 5,793914986 | 2,496011236 | 0,858383453 | 1,475205842 |
| **hsa-miR-376a-5p** | 0,701003491 | 3,947676061 | 2,697778851 | 0,000144262 | 0,616140144 |
| **hsa-miR-376b-3p** | 0,866942394 | 3,254684917 | 0,660872993 | 0,000144262 | 0,41465712 |
| **hsa-miR-376b-5p** | 0,807173611 | 2,844865318 | 1,217937181 | 0,000144262 | 0,000144262 |
| **hsa-miR-376c-5p** | 0,787177064 | 3,113840135 | 1,246153066 | 0,000144262 | 0,000144262 |
| **hsa-miR-377-5p** | 0,83019154 | 5,60607104 | 0,660872993 | 0,000144262 | 0,531202547 |
| **hsa-miR-380-3p** | 0,780661562 | 3,492302047 | 2,031062179 | 0,000144262 | 0,616140144 |
| **hsa-miR-3937** | 0,866419026 | 0,000144262 | 2,032110805 | 0,000144262 | 0,000144262 |
| **hsa-miR-3943** | 0,715523535 | 0,000144262 | 0,754246778 | 0,792817883 | 2,076589827 |
| **hsa-miR-410-5p** | 0,886139198 | 2,083890412 | 0,660872993 | 0,000144262 | 0,000144262 |
| **hsa-miR-412-5p** | 0,747835566 | 5,561784871 | 2,355028099 | 0,858383453 | 1,583707295 |
| **hsa-miR-431-3p** | 0,71422068 | 4,983087152 | 2,448646511 | 0,000144262 | 1,646520008 |
| **hsa-miR-432-5p** | 0,75746076 | 7,200330719 | 1,217937181 | 0,83517372 | 2,395460658 |
| **hsa-miR-433-5p** | 0,816073989 | 0,000144262 | 1,732857953 | 0,000144262 | 0,000144262 |
| **hsa-miR-4423-5p** | 0,730266781 | 1,097183874 | 0,000144262 | 0,83517372 | 1,873200655 |
| **hsa-miR-4483** | 0,801496264 | 0,668191606 | 2,910158562 | 0,000144262 | 0,000144262 |
| **hsa-miR-449b-5p** | 0,714414231 | 0,000144262 | 0,733891745 | 4,324906357 | 2,949779764 |
| **hsa-miR-4517** | 0,747357009 | 0,650519567 | 1,697140252 | 0,858383453 | 0,000144262 |
| **hsa-miR-4707-3p** | 0,845456944 | 0,650519567 | 0,000144262 | 0,000144262 | 0,000144262 |
| **hsa-miR-4725-3p** | 0,75213558 | 1,305999453 | 0,660872993 | 1,360669755 | 0,000144262 |
| **hsa-miR-487a-3p** | 0,836389704 | 4,245190247 | 0,660872993 | 0,792817883 | 0,918513358 |
| **hsa-miR-487a-5p** | 0,862755364 | 2,861174775 | 0,000144262 | 0,000144262 | 0,616140144 |
| **hsa-miR-493-3p** | 0,727767929 | 9,180698079 | 1,217937181 | 1,301476446 | 2,805699619 |
| **hsa-miR-496** | 0,737903115 | 2,958539833 | 2,254910484 | 0,000144262 | 0,458149299 |
| **hsa-miR-498** | 0,803436926 | 0,000144262 | 3,846973537 | 0,841932638 | 0,000144262 |
| **hsa-miR-5004-3p** | 0,738429433 | 0,000144262 | 0,660872993 | 0,922810582 | 1,223546004 |
| **hsa-miR-508-3p** | 0,718524549 | 0,000144262 | 1,189991938 | 3,144016752 | 1,996217762 |
| **hsa-miR-508-5p** | 0,828309554 | 0,000144262 | 0,714405852 | 0,922810582 | 0,531202547 |
| **hsa-miR-509-3p** | 0,715888438 | 0,000144262 | 1,547065794 | 2,196267108 | 1,583707295 |
| **hsa-miR-509-5p** | 0,817909199 | 0,000144262 | 0,42556153 | 2,907533995 | 1,084836566 |
| **hsa-miR-512-5p** | 0,846179459 | 0,000144262 | 2,53770733 | 0,000144262 | 0,000144262 |
| **hsa-miR-514a-5p** | 0,912421035 | 0,000144262 | 0,000144262 | 0,000144262 | 0,41465712 |
| **hsa-miR-515-5p** | 0,824686274 | 0,000144262 | 4,126301216 | 0,792817883 | 0,000144262 |
| **hsa-miR-516b-5p** | 0,717443641 | 0,000144262 | 6,904371792 | 2,340456435 | 0,458149299 |
| **hsa-miR-517-5p** | 0,881333626 | 0,000144262 | 1,732857953 | 0,000144262 | 0,000144262 |
| **hsa-miR-517a-3p** | 0,727431622 | 0,000144262 | 5,326939637 | 2,29864637 | 0,531202547 |
| **hsa-miR-517b-3p** | 0,727431622 | 0,000144262 | 5,326939637 | 2,29864637 | 0,531202547 |
| **hsa-miR-517c-3p** | 0,820085787 | 0,000144262 | 3,310045482 | 0,83517372 | 0,000144262 |
| **hsa-miR-518a-3p** | 0,881951615 | 0,000144262 | 2,356145028 | 0,000144262 | 0,000144262 |
| **hsa-miR-518b** | 0,749587215 | 0,000144262 | 5,016621482 | 1,370052327 | 0,531202547 |
| **hsa-miR-518c-3p** | 0,826857165 | 0,000144262 | 3,582067626 | 0,841932638 | 0,000144262 |
| **hsa-miR-518c-5p** | 0,802061828 | 0,000144262 | 3,901362864 | 0,792817883 | 0,000144262 |
| **hsa-miR-518e-3p** | 0,83353381 | 0,000144262 | 2,910158562 | 0,000144262 | 0,000144262 |
| **hsa-miR-518e-5p** | 0,837544196 | 0,000144262 | 2,619419458 | 0,792817883 | 0,000144262 |
| **hsa-miR-518f-5p** | 0,872216474 | 0,000144262 | 2,341038804 | 0,000144262 | 0,000144262 |
| **hsa-miR-519a-3p** | 0,812228809 | 0,000144262 | 2,84278035 | 0,858383453 | 0,000144262 |
| **hsa-miR-519a-5p** | 0,800253162 | 0,000144262 | 2,885658202 | 1,301476446 | 0,000144262 |
| **hsa-miR-519b-3p** | 0,853638688 | 0,000144262 | 3,206640714 | 0,000144262 | 0,000144262 |
| **hsa-miR-519b-5p** | 0,837544196 | 0,000144262 | 2,619419458 | 0,792817883 | 0,000144262 |
| **hsa-miR-519c-3p** | 0,859627594 | 0,000144262 | 2,448646511 | 0,000144262 | 0,000144262 |
| **hsa-miR-519c-5p** | 0,837544196 | 0,000144262 | 2,619419458 | 0,792817883 | 0,000144262 |
| **hsa-miR-519d-3p** | 0,804128641 | 0,000144262 | 5,071076558 | 0,83517372 | 0,000144262 |
| **hsa-miR-520a-3p** | 0,786935423 | 0,000144262 | 4,930627789 | 0,83517372 | 0,000144262 |
| **hsa-miR-520a-5p** | 0,817630303 | 0,000144262 | 4,10989484 | 0,000144262 | 0,000144262 |
| **hsa-miR-520c-3p** | 0,841263258 | 0,000144262 | 3,94541077 | 0,000144262 | 0,000144262 |
| **hsa-miR-520d-3p** | 0,844040813 | 0,000144262 | 2,950006805 | 0,000144262 | 0,000144262 |
| **hsa-miR-520d-5p** | 0,862671856 | 0,000144262 | 3,095248238 | 0,000144262 | 0,000144262 |
| **hsa-miR-520e** | 0,894430125 | 0,000144262 | 2,256007917 | 0,000144262 | 0,000144262 |
| **hsa-miR-520f-3p** | 0,766666421 | 0,000144262 | 5,687670921 | 0,83517372 | 0,000144262 |
| **hsa-miR-520g-3p** | 0,800041385 | 0,000144262 | 4,563022693 | 0,922810582 | 0,000144262 |
| **hsa-miR-520h** | 0,866969444 | 0,000144262 | 2,256007917 | 0,000144262 | 0,000144262 |
| **hsa-miR-521** | 0,803732294 | 0,000144262 | 2,256007917 | 0,000144262 | 0,000144262 |
| **hsa-miR-522-3p** | 0,711758362 | 0,000144262 | 3,977178433 | 1,781912374 | 0,531202547 |
| **hsa-miR-522-5p** | 0,837544196 | 0,000144262 | 2,619419458 | 0,792817883 | 0,000144262 |
| **hsa-miR-523-3p** | 0,908756563 | 0,000144262 | 1,765987066 | 0,000144262 | 0,000144262 |
| **hsa-miR-523-5p** | 0,837544196 | 0,000144262 | 2,619419458 | 0,792817883 | 0,000144262 |
| **hsa-miR-524-5p** | 0,823972576 | 0,000144262 | 3,008608928 | 0,000144262 | 0,000144262 |
| **hsa-miR-525-5p** | 0,836605764 | 0,000144262 | 3,096313733 | 0,000144262 | 0,000144262 |
| **hsa-miR-526a** | 0,875444362 | 0,000144262 | 1,904604061 | 0,000144262 | 0,000144262 |
| **hsa-miR-526b-3p** | 0,884073356 | 0,000144262 | 1,455711033 | 0,000144262 | 0,000144262 |
| **hsa-miR-526b-5p** | 0,717179672 | 0,000144262 | 5,874766492 | 2,088047528 | 0,616140144 |
| **hsa-miR-539-3p** | 0,720225409 | 6,725720852 | 0,754246778 | 1,676793207 | 2,114006588 |
| **hsa-miR-539-5p** | 0,824018284 | 3,974951032 | 0,660872993 | 0,000144262 | 0,531202547 |
| **hsa-miR-541-5p** | 0,963272223 | 1,868097737 | 0,000144262 | 0,000144262 | 0,000144262 |
| **hsa-miR-544b** | 0,781302695 | 0,000144262 | 0,000144262 | 0,000144262 | 0,000144262 |
| **hsa-miR-548ad** | 0,878646987 | 0,000144262 | 2,21620858 | 0,000144262 | 0,000144262 |
| **hsa-miR-548ao-3p** | 0,722619589 | 0,000144262 | 1,440992487 | 0,841932638 | 0,619583581 |
| **hsa-miR-549a** | 0,768821007 | 5,328145563 | 0,000144262 | 1,481045729 | 0,41465712 |
| **hsa-miR-5683** | 0,703852146 | 0,668191606 | 0,000144262 | 4,264847396 | 4,8725853 |
| **hsa-miR-573** | 0,733931644 | 0,000144262 | 1,994104402 | 1,360669755 | 0,000144262 |
| **hsa-miR-615-5p** | 0,710003235 | 2,784482696 | 0,000144262 | 1,974353102 | 0,619583581 |
| **hsa-miR-6500-3p** | 0,887869622 | 2,861174775 | 0,000144262 | 0,000144262 | 0,000144262 |
| **hsa-miR-6507-5p** | 0,777901717 | 0,000144262 | 0,000144262 | 2,310877694 | 0,41465712 |
| **hsa-miR-654-5p** | 0,74871923 | 7,801194215 | 0,42556153 | 0,83517372 | 2,786543684 |
| **hsa-miR-655-3p** | 0,780937305 | 5,60607104 | 0,754246778 | 0,000144262 | 1,583707295 |
| **hsa-miR-656-3p** | 0,730985452 | 6,582343034 | 0,660872993 | 0,858383453 | 2,041009142 |
| **hsa-miR-665** | 0,878634034 | 4,519226509 | 0,000144262 | 0,000144262 | 0,000144262 |
| **hsa-miR-6720-3p** | 0,755872525 | 2,615133925 | 0,000144262 | 0,841932638 | 0,458149299 |
| **hsa-miR-6840-5p** | 0,838732231 | 0,000144262 | 0,714405852 | 0,83517372 | 0,41465712 |
| **hsa-miR-876-3p** | 0,760285999 | 0,000144262 | 0,000144262 | 0,858383453 | 1,696959898 |
| **hsa-miR-888-5p** | 0,734681087 | 0,000144262 | 1,189991938 | 2,29864637 | 0,616140144 |
| **hsa-miR-934** | 0,763765432 | 0,000144262 | 1,731473442 | 8,042374411 | 0,458149299 |
| **hsa-miR-944** | 0,87376528 | 0,000144262 | 0,000144262 | 1,745028845 | 0,805256779 |
