# Supplementary material for: MicroRNA-934 is a novel primate-specific small non-coding RNA with neurogenic function during early development
Source: eLife. 2020 May 27;9:e50561. doi: 10.7554/eLife.50561 (PMC7295570; doi:10.7554/eLife.50561)
Supplement: Supplementary file 2. [file elife-50561-supp2.docx]

**Supplemental Table 2.** The 10 most highly expressed miRNAs in NPCs demonstrating tissue specificity index tau > 0.7. The miRNA name, tau index and expression values (in log2; reads per million) are shown. hsa-miR-934 ranks highest in NPC expression among the miRNAs with cell type specificity and is clearly segregated against all other stages. Warmer and cooler colors signify higher and smaller expression values, respectively.

| **miRNAs** | **Tau** | **Fibroblasts** | **iPSCs-ESCs** | **NPCs** | **Neurons** |
| --- | --- | --- | --- | --- | --- |
| hsa-miR-934 | 0.764 | 0.00014 | 1.73147 | 8.04237 | 0.45815 |
| hsa-miR-449b-5p | 0.714 | 0.00014 | 0.73389 | 4.32491 | 2.94978 |
| hsa-miR-5683 | 0.704 | 0.66819 | 0.00014 | 4.26485 | 4.87259 |
| hsa-miR-373-3p | 0.706 | 0.00014 | 6.48765 | 3.82604 | 0.00014 |
| hsa-miR-508-3p | 0.719 | 0.00014 | 1.18999 | 3.14402 | 1.99622 |
| hsa-miR-141-3p | 0.708 | 0.66819 | 3.65751 | 3.02348 | 0.80526 |
| hsa-miR-371a-3p | 0.757 | 0.00014 | 4.53010 | 3.01004 | 0.00014 |
| hsa-miR-509-5p | 0.818 | 0.00014 | 0.42556 | 2.90753 | 1.08484 |
| hsa-miR-183-3p | 0.719 | 0.00014 | 2.35615 | 2.86233 | 0.00014 |
| hsa-miR-1245a | 0.787 | 1.09718 | 0.00014 | 2.67227 | 0.61958 |
