# Supplementary material for: MicroRNA-934 is a novel primate-specific small non-coding RNA with neurogenic function during early development
Source: eLife. 2020 May 27;9:e50561. doi: 10.7554/eLife.50561 (PMC7295570; doi:10.7554/eLife.50561)
Supplement: Supplementary file 3. [file elife-50561-supp3.docx]

**Supplemental Table 3.** All Blastn results with evalue<1 for a query of the mature hsa-miR-934 sequence against all mature miRNAs in miRBase v22.1. Species, miRNA id and blastn match characteristics (query start-end, subject start-end, strand, score, evaule) are presented. All species detected by querying all sequences deposited in the dataset of mature miRNAs and miRNA precursors are primates (Homo sapiens, Pan troglodytes, Pongo pygmaeus, Macaca mulatta, Callithrix jacchus), providing further evidence of miR-934 being a primate specific miRNA.

| **Species** | **ID** | **Query start** | **Query end** | **Subject start** | **Subject end** | **Strand** | **Score** | **Evalue** |
| --- | --- | --- | --- | --- | --- | --- | --- | --- |
| **Homo sapiens** | hsa-miR-934 | 1 | 22 | 1 | 22 | + | 110 | 9.00E-04 |
| **Pan troglodytes** | ptr-miR-934 | 1 | 22 | 1 | 22 | + | 110 | 9.00E-04 |
| **Pongo pygmaeus** | ppy-miR-934 | 1 | 22 | 1 | 22 | + | 110 | 9.00E-04 |
| **Macaca mulatta** | mml-miR-934-5p | 1 | 21 | 1 | 21 | + | 105 | 0.002 |
| **Callithrix jacchus** | cja-miR-934 | 1 | 21 | 1 | 21 | + | 78 | 0.4 |
