# Supplementary material for: MicroRNA-934 is a novel primate-specific small non-coding RNA with neurogenic function during early development
Source: eLife. 2020 May 27;9:e50561. doi: 10.7554/eLife.50561 (PMC7295570; doi:10.7554/eLife.50561)
Supplement: Supplementary file 4. [file elife-50561-supp4.docx]

**Supplemental Table 4.** IDs and description of the small RNA-Seq human libraries of human tissue and cell data included in the analysis for miR934 expression. The accession IDs can be used to identify each sample and extract further information at the originating depository for raw sequencing data and alignment information from high-throughput sequencing experiments (Read Archive, SRA) at the URL link: <https://www.ncbi.nlm.nih.gov/sra>.

| **Sample ID** | **Sample description** | **Category** | **N (category)** | **miRNA** | **MIMAT** | **RPM** | **Category**  **Average RPM** |
| --- | --- | --- | --- | --- | --- | --- | --- |
| NES/present study | HUES6-NPCs | Neural induction Sample | 5 | hsa-miR-934 | MIMAT0004977 | 714.78 | 714.78 |
| SRR1636969 | hESC.H7 | Embryonic Stem Cells | 9 | hsa-miR-934 | MIMAT0004977 | 2.29667751 | 0.29 |
| SRR1636968 | hESC.H7 |  |  | hsa-miR-934 | MIMAT0004977 | 0.64513867 |  |
| SRR1636959 | hESC.H7 |  |  | hsa-miR-934 | MIMAT0004977 | 0 |  |
| SRR1636960 | hESC.H7 |  |  | hsa-miR-934 | MIMAT0004977 | 0 |  |
| SRR1636962 | hESC.H7 |  |  | hsa-miR-934 | MIMAT0004977 | 0 |  |
| SRR1636963 | hESC.H7 |  |  | hsa-miR-934 | MIMAT0004977 | 0 |  |
| SRR1636965 | hESC.H7 |  |  | hsa-miR-934 | MIMAT0004977 | 0 |  |
| SRR1988287 | hESC.H9. |  |  | hsa-miR-934 | MIMAT0004977 | 0 |  |
| SRR1988288 | hESC.H9. |  |  | hsa-miR-934 | MIMAT0004977 | 0 |  |
| SRR1988291 | D14 Differentiated forebrain cells from hESC.H9. | Forebrain Neurons | 2 | hsa-miR-934 | MIMAT0004977 | 0 | 0 |
| SRR1988292 | D14 Differentiated forebrain cells from hESC.H9. |  |  | hsa-miR-934 | MIMAT0004977 | 0 |  |
| Nowakowski, Rani et al. 2018 (sample 3)  SRR6328631 | Gestation Week 16.5 Primary Visual Cortex (V1) * | Cortex V1 GW16.5 | 1 | hsa-miR-934 | MIMAT0004977 | 3.65 | 3.65 |
| Nowakowski, Rani et al. 2018 (Sample 1)  SRR6328627 | Gestation Week 19.5 Primary Motor Cortex (M1) * | Cortex M1 GW19 | 1 | hsa-miR-934 | MIMAT0004977 | 4.1 | 4.1 |
| Nowakowski, Rani et al. 2018 Sample 2)  SRR6328628 | Gestation Week 19.5 Prefrontal Cortex (PFC) * | Prefrontal Cortex GW19.5 | 1 | hsa-miR-934 | MIMAT0004977 | 8.11 | 8.11 |
| Nowakowski, Rani et al. 2018 (Sample 4)  SRR6328630 | Gestation Week 19.5 Primary Motor Cortex (M1) * | Cortex V1 GW19.5 | 1 | hsa-miR-934 | MIMAT0004977 | 10.49 | 10.49 |
| SRR1658346 | CNS | Adult CNS | 4 | hsa-miR-934 | MIMAT0004977 | 0 | 0.03333516 |
| SRR2061800 | CNS |  |  | hsa-miR-934 | MIMAT0004977 | 0 |  |
| SRR2061801 | CNS |  |  | hsa-miR-934 | MIMAT0004977 | 0 |  |
| SRR1658360 | CNS |  |  | hsa-miR-934 | MIMAT0004977 | 0.13334065 |  |
| SRR1759212 | CNS.Brain prefrontal cortex (Brodmann Area 9) | Adult CNS Prefrontal Cortex | 2 | hsa-miR-934 | MIMAT0004977 | 0 | 0 |
| SRR1759213 | CNS.Brain prefrontal cortex (Brodmann Area 9) |  |  | hsa-miR-934 | MIMAT0004977 | 0 |  |
| SRR828708 | CNS.Temporal Neocortex Gray Matter Healthy | Adult Neocortex Gray Matter | 2 | hsa-miR-934 | MIMAT0004977 | 0 | 0 |
| SRR828709 | CNS.Temporal Neocortex Gray Matter Healthy |  |  | hsa-miR-934 | MIMAT0004977 | 0 |  |
| SRR531688 | CNS....Cancer.Germ cell tumor.Diseased | Germ cell tumor CNS | 2 | hsa-miR-934 | MIMAT0004977 | 4.1639674 | 5.24954232 |
| SRR531687 | CNS....Cancer.Germ cell tumor.Diseased |  |  | hsa-miR-934 | MIMAT0004977 | 6.33511725 |  |
| SRR531692 | CNS....Cancer.Glioma.Diseased | Glioma CNS | 2 | hsa-miR-934 | MIMAT0004977 | 0.2855791 | 0.14278955 |
| SRR531694 | CNS....Cancer.Glioma.Diseased |  |  | hsa-miR-934 | MIMAT0004977 | 0 |  |
| SRR531683 | CNS....Cancer.Embryonal tumor.Diseased | Embryonal tumor CNS | 2 | hsa-miR-934 | MIMAT0004977 | 0 | 0 |
| SRR531684 | CNS....Cancer.Embryonal tumor.Diseased |  |  | hsa-miR-934 | MIMAT0004977 | 0 |  |
| SRR1949839 | Whole blood | Blood | 8 | hsa-miR-934 | MIMAT0004977 | 0 | 0 |
| SRR1949841 | Whole blood |  |  | hsa-miR-934 | MIMAT0004977 | 0 |  |
| SRR1949847 | Whole blood |  |  | hsa-miR-934 | MIMAT0004977 | 0 |  |
| SRR1949850 | Whole blood |  |  | hsa-miR-934 | MIMAT0004977 | 0 |  |
| SRR1949858 | Whole blood |  |  | hsa-miR-934 | MIMAT0004977 | 0 |  |
| SRR1949861 | Whole blood |  |  | hsa-miR-934 | MIMAT0004977 | 0 |  |
| SRR2061795 | Whole blood |  |  | hsa-miR-934 | MIMAT0004977 | 0 |  |
| SRR2061797 | Whole blood |  |  | hsa-miR-934 | MIMAT0004977 | 0 |  |
| SRR2061803 | Heart | Heart | 2 | hsa-miR-934 | MIMAT0004977 | 0 | 0 |
| SRR2061804 | heart |  |  | hsa-miR-934 | MIMAT0004977 | 0 |  |
| SRR1820679 | muscle | Muscle | 2 | hsa-miR-934 | MIMAT0004977 | 0 | 0 |
| SRR1820680 | muscle |  |  | hsa-miR-934 | MIMAT0004977 | 0 |  |
| SRR2061810 | liver | liver | 1 | hsa-miR-934 | MIMAT0004977 | 0 | 0 |
| SRR2174513 | skin | Skin | 12 | hsa-miR-934 | MIMAT0004977 | 1.43524435 | 0.64598093 |
| SRR2174514 | skin |  |  | hsa-miR-934 | MIMAT0004977 | 1.36030336 |  |
| SRR2174515 | skin |  |  | hsa-miR-934 | MIMAT0004977 | 1.05325864 |  |
| SRR2174516 | skin |  |  | hsa-miR-934 | MIMAT0004977 | 0 |  |
| SRR2174517 | skin |  |  | hsa-miR-934 | MIMAT0004977 | 0 |  |
| SRR2174518 | skin |  |  | hsa-miR-934 | MIMAT0004977 | 0 |  |
| SRR2174519 | skin |  |  | hsa-miR-934 | MIMAT0004977 | 0.29220076 |  |
| SRR2174520 | skin |  |  | hsa-miR-934 | MIMAT0004977 | 0 |  |
| SRR2174537 | skin |  |  | hsa-miR-934 | MIMAT0004977 | 0 |  |
| SRR2174538 | skin |  |  | hsa-miR-934 | MIMAT0004977 | 0 |  |
| SRR2174541 | skin |  |  | hsa-miR-934 | MIMAT0004977 | 0 |  |
| SRR2174542 | skin |  |  | hsa-miR-934 | MIMAT0004977 | 3.61076402 |  |
| SRR191548 | breast | Breast | 2 | hsa-miR-934 | MIMAT0004977 | 73.5042843 | 50.8485084 |
| SRR191578 | breast |  |  | hsa-miR-934 | MIMAT0004977 | 28.1927326 |  |
| SRR070232 | kidney | Kidney | 2 | hsa-miR-934 | MIMAT0004977 | 1.49839432 | 0.99601097 |
| SRR070230 | kidney |  |  | hsa-miR-934 | MIMAT0004977 | 0.49362762 |  |

* high-throughput sequencing of small RNA isolated by crosslinking immunoprecipitation (HITS-CLIP) with AGO2 in primary
developing human brain tissues.
