# Supplementary material for: MicroRNA-934 is a novel primate-specific small non-coding RNA with neurogenic function during early development
Source: eLife. 2020 May 27;9:e50561. doi: 10.7554/eLife.50561 (PMC7295570; doi:10.7554/eLife.50561)
Supplement: Supplementary file 5. [file elife-50561-supp5.docx]

**Supplemental Table 5.** Identification of all predicted mRNA targets for miRNA-934 by exploring the RNA-seq data obtained upon transition from hESCs/iPSCs to NPCs, as well as a second set of RNA-seq data generated at the stage of neural induction of hESCs following sustained inhibition of miR-934. In each case the analysis included integration of small RNA and RNA sequencing data using the algorithm presented in mirExTra v2. Using the microT-CDS target prediction tool as the source of potential interactions and applying a microT-CDS prediction threshold of 0.7 the indicated predicted targets were identified.

| **miR-934 targets identified upon the transition**  **from hESCs/iPSCs to NPCs** | **miR-934 targets identified following sustained inhibition of miR-934 function at the NPCs stage** |
| --- | --- |
| F11R (microT-CDS prediction score: 0.991) | STMN2 (microT-CDS prediction score: 0.853) |
| SLC16A1 (microT-CDS prediction score: 0.989) | TFCP2L1 (microT-CDS prediction score: 0.796) |
| FZD5 (microT-CDS prediction score: 0.851) | RAB3B (microT-CDS prediction score: 0.755) |
