# Supplementary material for: MicroRNA-934 is a novel primate-specific small non-coding RNA with neurogenic function during early development
Source: eLife. 2020 May 27;9:e50561. doi: 10.7554/eLife.50561 (PMC7295570; doi:10.7554/eLife.50561)
Supplement: Supplementary file 6. [file elife-50561-supp6.docx]

**Supplemental Table 6.** 3’ UTR binding sites for miR934 on its predicted targets (adapted from Diana microT-CDS)

| **Target** | **Binding Type** | **Score** | **Position on chromosome** | **Conserved Species** | **Binding Area** |
| --- | --- | --- | --- | --- | --- |
| **STMN2** | 8mer | 0.039 | 8:80577484-80577512 | panTro2,rheMac2  rn4,mm9,oryCun2  bosTau4,canFam2  dasNov2,loxAfr3 | \| (Transcript) \| 5' \| ______AAAGAAAAAUGAG__C________ \| 3' \|  \|  \|  \|  \| \| --- \| --- \| --- \| --- \| --- \| --- \| --- \| --- \| \|  \|  \| _UUCCA_____________GU_AGUAGACA \|  \|  \|  \|  \|  \| \|  \|  \| _.\|\|\|\|_____________\|\|_\|\|\|\|\|\|\|\| \|  \|  \|  \|  \|  \| \|  \|  \| _GAGGU_____________CA_UCAUCUGU \|  \|  \|  \|  \|  \| \| (miRNA) \| 3' \| A_____________________________ \| 5' \|  \|  \|  \|  \| |
| **TFCP2L1** | 7mer | 0.01 | 2:121981854-121981879 | panTro2  monDom5 | \| (Transcript) \| 5' \| AGC__U___GA___CC___GAU_______ \| 3' \| \| --- \| --- \| --- \| --- \| \|  \|  \| ___CC_GUG__UCC__GUG___GUAGACA \|  \| \|  \|  \| ___\|\|_\|\|\|__\|\|\|__\|\|.___\|\|\|\|\|\|\| \|  \| \|  \|  \| ___GG_CAC__AGG__CAU___CAUCUGU \|  \| \| (miRNA) \| 3' \| _____U___AG___U______________ \| 5' \| |
|  | 9mer | 0.02 | 2:121975850-121975867 | panTro2,rheMac2 | \| (Transcript) \| 5' \| AAAAAAAAAAU____AUUUU_________ \| 3' \| \| --- \| --- \| --- \| --- \| \|  \|  \| ___________UCUU_____UAGUAGACA \|  \| \|  \|  \| ___________\|\|\|._____\|\|\|\|\|\|\|\|\| \|  \| \|  \|  \| ___________AGAG_____AUCAUCUGU \|  \| \| (miRNA) \| 3' \| __________C____GUC___________ \| 5' \| |
| **RAB3B** | 7mer | 0.006 | 1:52376808-52376829 | panTro2 | \| (Transcript) \| 5' \| UGGCUUC_______A_____CA________ \| 3' \| \| --- \| --- \| --- \| --- \| \|  \|  \| _______CCAG_GU_UCCAG__GGUAGACA \|  \| \|  \|  \| _______\|\|\|\|_\|\|_\|\|\|\|\|__.\|\|\|\|\|\|\| \|  \| \|  \|  \| _______GGUC_CA_AGGUC__UCAUCUGU \|  \| \| (miRNA) \| 3' \| ___________A__G_____A_________ \| 5' \| |
|  | 7mer | 0.002 | 1:52375870-52375892 | Not Conserved | \| (Transcript) \| 5' \| AGUAAA_________U___ACCCCU_______ \| 3' \| \| --- \| --- \| --- \| --- \| \|  \|  \| ______CUA___UCU_CCA______GUAGACA \|  \| \|  \|  \| ______\|.\|___\|\|\|_\|\|\|______\|\|\|\|\|\|\| \|  \| \|  \|  \| ______GGU___AGA_GGU______CAUCUGU \|  \| \| (miRNA) \| 3' \| _________CAC_______CAU__________ \| 5' \| |
|  | 8mer | 0.01 | 1:52380265-52380293 | panTro2 | \| (Transcript) \| 5' \| _______CUGA____UUAUAU_A________ \| 3' \| \| --- \| --- \| --- \| --- \| \|  \|  \| _AG_GUC____UCCA______G_AGUAGACA \|  \| \|  \|  \| _\|\|_\|\|\|____\|\|\|\|______\|_\|\|\|\|\|\|\|\| \|  \| \|  \|  \| _UC_CAG____AGGU______C_UCAUCUGU \|  \| \| (miRNA) \| 3' \| G__A__________________A________ \| 5' \| |
|  | 8mer | 0.01 | 1:52380265-52380293 | panTro2 | \| (Transcript) \| 5' \| _______CUGA____UUAUAU_A________ \| 3' \| \| --- \| --- \| --- \| --- \| \|  \|  \| _AG_GUC____UCCA______G_AGUAGACA \|  \| \|  \|  \| _\|\|_\|\|\|____\|\|\|\|______\|_\|\|\|\|\|\|\|\| \|  \| \|  \|  \| _UC_CAG____AGGU______C_UCAUCUGU \|  \| \| (miRNA) \| 3' \| G__A__________________A________ \| 5' \| |
| **FZD5** | 7mer | 0.03 | 2:208627688-208627711 | panTro2,rheMac2rn4,mm9,oryCun2bosTau4,canFam2loxAfr3 | \| (Transcript) \| 5' \| UAGAG____C_AUA_____UUU_______ \| 3' \| \| --- \| --- \| --- \| --- \| \|  \|  \| _____CCAG_G___CUUUA___GUAGACA \|  \| \|  \|  \| _____\|\|\|\|_\|___\|\|..\|___\|\|\|\|\|\|\| \|  \| \|  \|  \| _____GGUC_C___GAGGU___CAUCUGU \|  \| \| (miRNA) \| 3' \| _________A_A_______CAU_______ \| 5' \| |
|  | 8mer | 0.005 | 2:208629524-208629544 | panTro2 | \| (Transcript) \| 5' \| AUUUCAUA_____UC____AA________ \| 3' \| \| --- \| --- \| --- \| --- \| \|  \|  \| ________UGUCU__CCAG__AGUAGACA \|  \| \|  \|  \| ________\|\|\|\|\|__\|\|\|\|__\|\|\|\|\|\|\|\| \|  \| \|  \|  \| ________ACAGA__GGUC__UCAUCUGU \|  \| \| (miRNA) \| 3' \| _______C___________A_________ \| 5 \| |
