# Supplementary material for: MicroRNA-934 is a novel primate-specific small non-coding RNA with neurogenic function during early development
Source: eLife. 2020 May 27;9:e50561. doi: 10.7554/eLife.50561 (PMC7295570; doi:10.7554/eLife.50561)
Supplement: Supplementary file 7. [file elife-50561-supp7.docx]

**Supplemental Table 7.** Sequences of primers and oligos.

| **PRIMERS** | **SEQUENCE (5'-3')** |
| --- | --- |
| GAPDH forward primer: | CCTCTGACTTCAACAGCGACAC |
| GAPDH reverse primer: | AGCCAAATTCGTTGTCATACCAG |
| PAX6 forward primer: | TGTCCAACGGATGTGTGAGT |
| PAX6 reverse primer: | TTTCCCAAGCAAAGATGGAC |
| DCX forward primer: | TCAGGGAGTGCGTTACATTTAC |
| DCX reverse primer: | GTTGGGATTGACATTCTTGGTG |
| FZD5 forward primer: | TGTCTGCTCTTCTCGGC' |
| FZD5 reverse primer: | CCGTCCAAAGATAAACTGCT |
| STMN2 forward primer: | CTTGAAGCCACCATCTCCTATC |
| STMN2 reverse primer: | GCCTCCTGAGACTTTCTTCTTT |
| TFCP2L1 forward primer: | GTCCAACCCTGACGTTCTAAA |
| TFCP2L1 reverse primer: | ATACAGGCAGCACCAAGATAC |
| RAB3B forward primer: | CTTGGCCACAGGGACATATT |
| RAB3B reverse primer: | CTGAGGATTCAGGCCATGATAG |
| LEF1 forward primer: | CTTGTCTGGTAAGTGGCTTCTC |
| LEF1 reverse primer: | ACAGAGTGGGTTTGGCTATTAC |
| TFAP2C forward primer: | CGGGAGAAGTTGGACAAGATTG |
| TFAP2C reverse primer: | ATTCGGCTTCACAGACATAGG |
| MOXD1 forward primer: | TCTTCCATACCATCCCTCCAG |
| MOXD1 reverse primer: | ACAGCAAACACATGAATTCCAC |
| ID2 forward primer: | CAAGAAGGTGAGCAAGATGGA |
| ID2 reverse primer: | GGTGATGCAGGCTGACAATA |
| NCAD forward primer: | CCCAAGACAAAGAGACCCAG |
| NCAD reverse primer: | GCCACTGTGCTTACTGAATTG |
| GATA2 forward primer: | GACGACAACCACCACCTTAT |
| GATA2 reverse primer: | AGTCTGGATCCCTTCCTTCT |
| SYNGAP1 forward primer: | GGGTTTAGGAGGGTGTGTATG |
| SYNGAP1 reverse primer: | GGGAGTAGAGACCAAGAAGAGA |
| PRSS12 forward primer: | CCACAGAAAACAGCATCCAAC |
| PRSS12 reverse primer: | AGCATTCTCCCTGTAAACCG |
| TPD52L1 forward primer: | TGTTGGAGACTGAACCGTTG |
| TPD52L1 reverse primer: | CTTGTCGTAGTGTTGTAATTTCGTC |
| Reverse primer R1 | **TGCTCTAGA**CCCCTAAGCTATGAAGTGAGGC |
| Forward primer F2 | **TAAAGAGCTC**GAGCTGCGCTAATCCTGACA |
| Reverse primer R2 | **TGCTCTAGA**ACTAACATAACCACATACAACTGCT |
| Forward primer F3 | **CTAGCTAGC**GAAGAAGCCTCTTCCTAGAATC |
| Forward primer F4 | **ATCGAGCTC**CTGAGAGCAATGATGGCTACCA |
| Reverse primer R4 | **TGCTCTAGA**GCTGGTTGGTGCTCTGTAGCTTT |
| Forward primer F5 | **ATCGAGCTC**CTCATCCTGTATCAGCACTTACC |
| Reverse primer R5 | **TGCTCTAGA**CGGTAAATATTTTGGGCTTTCAGAGC |
| Forward primer F6 | **ATCGAGCTC**GATCTCATTGATTTCAGATTCCC |
| Reverse primer R6 | **TGCTCTAGA**GGTGATGATTTGACAATGTCTATAC |
| Forward primer F7 | **ATCGAGCTC**AGAAATGCATTCCACCTCAAGGA |
| Reverse primer R7 | **TGCTCTAGA**ACATATGAAGTAACTGAGGCTCAC |
| Forward primer FOR MUT | TTTTATTTTACATGTATTTACCAAAAATATGTACT |
| Reverse primer REV MUT | ATAAGAATTTTTTTTTTTGTGATGGGGG |
| **OLIGOS** | **SEQUENCE** |
| Fzd5 8-mer MRE Sense 5'- 3' | CTAGCTAGCGGCCGCTAGTATTTCATATGTCTTCCCAGAAAGTAGACAT |
| Fzd5 8-mer MRE Anti-sense: 5'-3' | CTAGATGTCTACTTTCTGGGAAGACATATGAAATACTAGCGGCCGCTAG |
| mutated Fzd5 8-mer MRE Sense 5'- 3' | CTAGCTAGCGGCCGCTAGTATTTCATATGTCTTCCCAGAATTTTTTTT |
| mutated Fzd5 8-mer anti-sense: 5'-3' | CTAGAAAAAAAAATTCTGGGAAGACATATGAAATACTAGCGGCCGCTAG |
