# Supplementary material for: MicroRNA-934 is a novel primate-specific small non-coding RNA with neurogenic function during early development
Source: eLife. 2020 May 27;9:e50561. doi: 10.7554/eLife.50561 (PMC7295570; doi:10.7554/eLife.50561)
Supplement: Supplementary file 8. [file elife-50561-supp8.docx]

**Supplemental Table 8.** Significance (P value) of differential expression, description and related references of progenitor-specific and SP-enriched genes affected by sustained inhibition of miR-934 and depicted in heatmap on Fig. 5F.

| **GENE** | **P-VALUE** | **DESCRIPTION** | **REFERENCE(S)** |
| --- | --- | --- | --- |
| **TFAP2C** | 6,13E-07 | Dorsal radial glia/apical progenitors | Pinto L, Drechsel D, Schmid MT, Ninkovic J, Irmler M, Brill MS, Restani L, Gianfrancheschi L, Cerri C, Weber SN, et.al. 2009. AP2gamma regulates basal progenitor fate in a region- and layer-specific manner in the developing cortex. Nat. Neurosci. 12(10):1229-37.  Nowakowski TJ, Bhaduri A, Pollen AA, Alvarado B, Mostajo-Radji MA, Di Lullo E, Haeussler M, Sandoval-Espinosa C, Liu SJ, Velmeshev D, Ounadjela JR, et.al. 2017. Spatiotemporal gene expression trajectories reveal developmental hierarchies of the human cortex. Science 358(6368):1318-1323. |
| **LEF1** | 0,005107 | Early cortical progenitors | Nowakowski TJ, Bhaduri A, Pollen AA, Alvarado B, Mostajo-Radji MA, Di Lullo E, Haeussler M, Sandoval-Espinosa C, Liu SJ, Velmeshev D, Ounadjela JR, et.al. 2017. Spatiotemporal gene expression trajectories reveal developmental hierarchies of the human cortex. Science 358(6368):1318-1323.  Gan Q, Lee A, Suzuki R, Yamagami T, Stokes A, Nguyen BC, Pleasure D, Wang J, Chen HW, Zhou CJ, 2014. Pax6 mediates ß-catenin signaling for self-renewal and neurogenesis by neocortical radial glial stem cells. Stem Cells **32(1):45-58.** |
| **MOXD1** | 9,53E-17 | Dorsal radial glia | Nowakowski TJ, Bhaduri A, Pollen AA, Alvarado B, Mostajo-Radji MA, Di Lullo E, Haeussler M, Sandoval-Espinosa C, Liu SJ, Velmeshev D, Ounadjela JR, et.al. 2017. Spatiotemporal gene expression trajectories reveal developmental hierarchies of the human cortex. Science 358(6368):1318-1323. |
| **TPD52L1** | 8,32E-06 | Subplate-enriched | Luhmann HJ, Kirischuk S, Kilb W, 2018. The Superior Function of the Subplate in Early Neocortical Development. Front Neuroanat. 12:97.  Hoerder-Suabedissen A, Oeschger FM, Krishnan ML, Belgrad TG, Wang WZ, Lee S, Webber C, Petretto E, Edwards AD, Molnar Z, 2013. Expression profiling of mouse subplate reveals a dynamic gene network and disease association with autism and schizophrenia. Proc Natl Acad Sci USA 110(9):3555-60. |
| **CADPS2** | 0,0001 | Subplate-enriched | Miller JA, Ding SL, Sunkin SM, Smith KA, Ng L, Szafer A, Ebbert A, Riley ZL, Royall JJ, Aiona K, et.al. 2014. Transcriptional landscape of the prenatal human brain. Nature 508(7495):199-206.  Hoerder-Suabedissen A, Oeschger FM, Krishnan ML, Belgrad TG, Wang WZ, Lee S, Webber C, Petretto E, Edwards AD, Molnar Z, 2013. Expression profiling of mouse subplate reveals a dynamic gene network and disease association with autism and schizophrenia. Proc Natl Acad Sci USA 110(9):3555-60. |
| **CDH18** | 0,003904 | Subplate-enriched | Miller JA, Ding SL, Sunkin SM, Smith KA, Ng L, Szafer A, Ebbert A, Riley ZL, Royall JJ, Aiona K, et.al. 2014. Transcriptional landscape of the prenatal human brain. Nature 508(7495):199-206.  Hoerder-Suabedissen A, Oeschger FM, Krishnan ML, Belgrad TG, Wang WZ, Lee S, Webber C, Petretto E, Edwards AD, Molnar Z, 2013. Expression profiling of mouse subplate reveals a dynamic gene network and disease association with autism and schizophrenia. Proc Natl Acad Sci USA 110(9):3555-60. |
| **PRSS12** | 1,42E-07 | Subplate-enriched | Hoerder-Suabedissen A, Oeschger FM, Krishnan ML, Belgrad TG, Wang WZ, Lee S, Webber C, Petretto E, Edwards AD, Molnar Z, 2013. Expression profiling of mouse subplate reveals a dynamic gene network and disease association with autism and schizophrenia. Proc Natl Acad Sci USA 110(9):3555-60.  Hoerder-Suabedissen A, and Molnar Z, 2015. Development, evolution and pathology of neocortical subplate neurons. Nat Rev Neurosci 16(3):133-46. |
| **SEMA5A** | 0,03374 | Subplate-enriched | Miller JA, Ding SL, Sunkin SM, Smith KA, Ng L, Szafer A, Ebbert A, Riley ZL, Royall JJ, Aiona K, et.al. 2014. Transcriptional landscape of the prenatal human brain. Nature 508(7495):199-206.  Hoerder-Suabedissen A, Oeschger FM, Krishnan ML, Belgrad TG, Wang WZ, Lee S, Webber C, Petretto E, Edwards AD, Molnar Z, 2013. Expression profiling of mouse subplate reveals a dynamic gene network and disease association with autism and schizophrenia. Proc Natl Acad Sci USA 110(9):3555-60. |
| **CDH10** | 6,13E-07 | Subplate-enriched | Miller JA, Ding SL, Sunkin SM, Smith KA, Ng L, Szafer A, Ebbert A, Riley ZL, Royall JJ, Aiona K, et.al. 2014. Transcriptional landscape of the prenatal human brain. Nature 508(7495):199-206.  Hoerder-Suabedissen A, Oeschger FM, Krishnan ML, Belgrad TG, Wang WZ, Lee S, Webber C, Petretto E, Edwards AD, Molnar Z, 2013. Expression profiling of mouse subplate reveals a dynamic gene network and disease association with autism and schizophrenia. Proc Natl Acad Sci USA 110(9):3555-60. |
| **BACE2** | 0,009952 | Subplate-enriched | Miller JA, Ding SL, Sunkin SM, Smith KA, Ng L, Szafer A, Ebbert A, Riley ZL, Royall JJ, Aiona K, et.al. 2014. Transcriptional landscape of the prenatal human brain. Nature 508(7495):199-206. |
| **FAM43B** | 0,000574 | Subplate-enriched | Miller JA, Ding SL, Sunkin SM, Smith KA, Ng L, Szafer A, Ebbert A, Riley ZL, Royall JJ, Aiona K, et.al. 2014. Transcriptional landscape of the prenatal human brain. Nature 508(7495):199-206. |
| **GPD1L** | 0,000308 | Subplate-enriched | Miller JA, Ding SL, Sunkin SM, Smith KA, Ng L, Szafer A, Ebbert A, Riley ZL, Royall JJ, Aiona K, et.al. 2014. Transcriptional landscape of the prenatal human brain. Nature 508(7495):199-206. |
| **HAS3** | 1,06E-09 | Subplate-enriched | Miller JA, Ding SL, Sunkin SM, Smith KA, Ng L, Szafer A, Ebbert A, Riley ZL, Royall JJ, Aiona K, et.al. 2014. Transcriptional landscape of the prenatal human brain. Nature 508(7495):199-206. |
| **HS3ST4** | 0,00078 | Subplate-enriched | Miller JA, Ding SL, Sunkin SM, Smith KA, Ng L, Szafer A, Ebbert A, Riley ZL, Royall JJ, Aiona K, et.al. 2014. Transcriptional landscape of the prenatal human brain. Nature 508(7495):199-206. |
| **IGFBP3** | 6,54E-13 | Subplate-enriched | Miller JA, Ding SL, Sunkin SM, Smith KA, Ng L, Szafer A, Ebbert A, Riley ZL, Royall JJ, Aiona K, et.al. 2014. Transcriptional landscape of the prenatal human brain. Nature 508(7495):199-206. |
| **MICAL2** | 0,011806 | Subplate-enriched | Miller JA, Ding SL, Sunkin SM, Smith KA, Ng L, Szafer A, Ebbert A, Riley ZL, Royall JJ, Aiona K, et.al. 2014. Transcriptional landscape of the prenatal human brain. Nature 508(7495):199-206. |
| **PCDH1** | 0,00012 | Subplate-enriched | Miller JA, Ding SL, Sunkin SM, Smith KA, Ng L, Szafer A, Ebbert A, Riley ZL, Royall JJ, Aiona K, et.al. 2014. Transcriptional landscape of the prenatal human brain. Nature 508(7495):199-206. |
| **SEMA3E** | 3,21E-12 | Subplate-enriched | Miller JA, Ding SL, Sunkin SM, Smith KA, Ng L, Szafer A, Ebbert A, Riley ZL, Royall JJ, Aiona K, et.al. 2014. Transcriptional landscape of the prenatal human brain. Nature 508(7495):199-206. |
| **SORCS1** | 0,010331 | Subplate-enriched | Miller JA, Ding SL, Sunkin SM, Smith KA, Ng L, Szafer A, Ebbert A, Riley ZL, Royall JJ, Aiona K, et.al. 2014. Transcriptional landscape of the prenatal human brain. Nature 508(7495):199-206. |
| **SPSB1** | 0,042657 | Subplate-enriched | Miller JA, Ding SL, Sunkin SM, Smith KA, Ng L, Szafer A, Ebbert A, Riley ZL, Royall JJ, Aiona K, et.al. 2014. Transcriptional landscape of the prenatal human brain. Nature 508(7495):199-206. |
| **TRPM3** | 0,005671 | Subplate-enriched | Miller JA, Ding SL, Sunkin SM, Smith KA, Ng L, Szafer A, Ebbert A, Riley ZL, Royall JJ, Aiona K, et.al. 2014. Transcriptional landscape of the prenatal human brain. Nature 508(7495):199-206. |
| **SYT6** | 0,037786 | Subplate-enriched | Miller JA, Ding SL, Sunkin SM, Smith KA, Ng L, Szafer A, Ebbert A, Riley ZL, Royall JJ, Aiona K, et.al. 2014. Transcriptional landscape of the prenatal human brain. Nature 508(7495):199-206. |
